# Presentation to Dr. J. G. Swayne

**Published:** 1895-03

**Authors:** 


### Presentation to Dr. J. G. Swayne.

A very interesting event took place at the Clifton Down Hotel on the evening of February 20th, when about sixty medical men sat down to a complimentary dinner, given to Dr. J. G. Swayne in commemoration of the jubilee of his connection with the Bristol Medical School.

In October last Dr. Swayne terminated his active work at the school, having occupied the post of Lecturer on Midwifery for fifty years, and the occasion seemed a fitting one for emphasising such a remarkably long and eminent service by some token of regard and esteem. A committee, consisting of Mr. J. Paul Bush, Dr. J. Michell Clarke, Dr. A. J. Harrison, Dr. E. Markham Skerritt, and Dr. R. Shingleton Smith, soon succeeded in enlisting the support of a large number of the profession in Bristol and the neighbourhood, enabling them to procure a handsome service of plate which was to be presented at the dinner.

Dr. E. Long Fox presided, and after dinner Dr. Henry Marshall, in well chosen sentences, proposed the health of the guest of the evening, alluding to the circumstance that the date marked not only the jubilee of Dr. Swayne's lectureship, but also the jubilee of his graduation as Doctor of Medicine at London University. He spoke of the success of Swayne's *Aphorisms*, and also of the esteem with which Dr. Swayne was universally regarded. Allusion was also made to the liberality which Dr. Swayne displayed at the time of erecting the new Medical School buildings, and to the amount of time and work which he had continually given to the school during a long and busy private and hospital practice.

Mr. Arthur W. Prichard, in the absence of his father (Mr. Augustin Prichard), who was prevented by indisposition from being present, spoke in support of the toast.

Mr. Nelson C. Dobson added a few words, alluding chiefly to Dr. Swayne's work in connection with the local branch of the British Medical Association.

Dr. Markham Skerritt read letters from several who were unable to be present, and then mentioned that a few hours before the dinner the Council of University College, Bristol, had conferred the title of Emeritus Professor of Midwifery upon Dr. Swayne. He read a list of the subscribers to the testimonial, which was produced for the inspection of the guests. It consisted of a large silver tray, a bowl, and a pair of loving cups, all bearing Dr. Swayne's arms and crest. On the tray was the following inscription:

"Presented, with a service of plate, to Joseph Griffiths Swayne, M.D., Emeritus Professor of Midwifery in University College, Bristol, by his colleagues, friends, and former pupils, as a mark of their personal esteem, and in grateful recognition of his eminent services in the teaching of obstetric medicine and gynæcology in the Bristol Medical School and University College, Bristol, during the long period of fifty years. February 20, 1895."

On the back were engraved the names of the donors.

Dr. Long Fox in a few graceful words made the presentation, and the toast was drunk with great enthusiasm.

Dr. Swayne returned thanks, and alluded to the fact that his little work, *Obstetric Aphorisms*, reached its tenth edition in 1893, and that altogether 20,000 copies had been published. It had also been translated into eight languages, one of which was Hindustani.

